# Unfolded Protein Response (UPR) Regulator Cib1 Controls Expression of Genes Encoding Secreted Virulence Factors in *Ustilago maydis*

**DOI:** 10.1371/journal.pone.0153861

**Published:** 2016-04-19

**Authors:** Martin Hampel, Mareike Jakobi, Lara Schmitz, Ute Meyer, Florian Finkernagel, Gunther Doehlemann, Kai Heimel

**Affiliations:** 1 Department of Molecular Microbiology and Genetics, Institute for Microbiology and Genetics, Göttingen, Germany; 2 Center for Molecular Biosciences (GZMB), Georg-August-University, Göttingen, Germany; 3 Botanical Institute and Cluster of Excellence on Plant Sciences, University of Cologne, Cologne Germany; 4 Institute of Molecular Biology and Tumor Research (IMT), Philipps-University, Marburg, Germany; Hans-Knoell-Institute (HKI), GERMANY

## Abstract

The unfolded protein response (UPR), a conserved eukaryotic signaling pathway to ensure protein homeostasis in the endoplasmic reticulum (ER), coordinates biotrophic development in the corn smut fungus *Ustilago maydis*. Exact timing of UPR activation is required for virulence and presumably connected to the elevated expression of secreted effector proteins during infection of the host plant *Zea mays*. In the baker’s yeast *Saccharomyces cerevisiae*, expression of UPR target genes is induced upon binding of the central regulator Hac1 to unfolded protein response elements (UPREs) in their promoters. While a role of the UPR in effector secretion has been described previously, we investigated a potential UPR-dependent regulation of genes encoding secreted effector proteins. *In silico* prediction of UPREs in promoter regions identified the previously characterized effector genes *pit2* and *tin1-1*, as *bona fide* UPR target genes. Furthermore, direct binding of the Hac1-homolog Cib1 to the UPRE containing promoter fragments of both genes was confirmed by quantitative chromatin immunoprecipitation (qChIP) analysis. Targeted deletion of the UPRE abolished Cib1-dependent expression of *pit2* and significantly affected virulence. Furthermore, ER stress strongly increased Pit2 expression and secretion. This study expands the role of the UPR as a signal hub in fungal virulence and illustrates, how biotrophic fungi can coordinate cellular physiology, development and regulation of secreted virulence factors.

## Introduction

The infection process of plant pathogenic fungi and their host plants requires effective strategies to subvert plant defense responses and foster pathogenic growth. Typically, genes associated with host infection are transcriptionally induced upon entering the host plant [1–3]. While there is a growing body of information on effector protein function, regulation of effector gene expression is still poorly understood. In various plant-colonizing fungi effector gene expression is regulated on chromatin level [4,5], or is aligned to specific developmental stages and/or the different host environments [6–8].

The genome of the corn smut fungus *Ustilago maydis* contains 536 genes, which are predicted to encode secreted proteins that might function as effectors [1]. Many of these putative effector-encoding genes are organized in clusters and are highly upregulated during plant colonization [9]. Expression of genes encoding Pit2 and Pep1 effectors is transcriptionally induced already during epidermal infection, which is consistent with their crucial functions in establishing a compatible interaction with the host [10–14]. In contrast, other effectors including See1 and Tin2 show a different expression pattern with highest induction at later stages during tumorigenesis [15,16]. The concerted upregulation of effector gene expression imposes stress on the secretory pathway, which is counteracted by the unfolded protein response (UPR) pathway to restore homeostasis of the endoplasmic reticulum (ER) [17]. The UPR is a highly conserved eukaryotic signaling pathway coordinated by the ER membrane-localized kinase/RNase Ire1 and the bZIP transcription factor termed Hac1 in *Saccharomyces cerevisiae* and XBP1 in higher eukaryotes [18–20]. ER stress is manifested by accumulation of un- or misfolded proteins in the ER that are sensed by the ER luminal domain of Ire1 resulting in multimerization, trans-autophosphorylation, activation of the endoribonuclease domain and subsequent cleavage of the unconventional intron of the Hac1 encoding mRNA [21]. This process, referred to as unconventional cytoplasmic splicing gives rise to the Hac1 transcription factor, which subsequently induces UPR target gene expression by binding to unfolded protein response elements (UPREs) in their promoters [22–25]. ER homeostasis is restored by increasing the capacity for protein folding in the ER, by ER membrane expansion and by targeted degradation of irreversibly misfolded proteins [26]. Accordingly, UPR target genes typically encode ER chaperones, proteins involved in phospholipid and fatty acid synthesis and the ER associated degradation (ERAD) pathway [27].

In *U*. *maydis*, the UPR is adapted to the pathogenic lifestyle of the fungus. Similar to other plant pathogenic fungi, the UPR is required for pathogenicity of *U*. *maydis* but is in addition tightly connected to the regulatory pathways that control pathogenic development [28–31]. The *b* mating-type locus encoded bE/bW-heterodimer represents the central regulator of pathogenic development and triggers a dimorphic transition that is crucial for plant infection [32,33]. However, fungal proliferation after penetration of the host requires modification of the b-regulatory network by the Clp1 protein [34,35]. The UPR pathway is regulated by the Hac1 homologue Cib1 and specifically activated after successful host plant penetration [34]. Cib1 expression leads to stabilization of Clp1 and thereby promotes fungal proliferation in the host plant. It is thus conceivable that plant-specific UPR activation after host penetration facilitates developmental progression. By contrast, premature UPR activation suppresses expression of *bE* and *bW*, which leads to reduced filamentation and virulence. In addition, a functional UPR appears to be important for efficient secretion of effector proteins. Maize plants inoculated with Δ*cib1* strains showed increased *pathogen related* (*pr*)-gene expression [31] and secretion of the chorismate mutase Cmu1 under ER stress is dependent on the UPR-regulated ER co-chaperone Dnj1 [36]. Increased demands on the secretory pathway by upregulated expression of effector genes are assumed to activate the UPR pathway [31,36]. However, the multilayered crosstalk of UPR and regulatory pathways that control pathogenic development, prompted us to ask whether the role of the UPR is restricted to secretion of effectors, or might also affect transcriptional regulation of effector encoding genes.

Here we investigated a potential transcriptional regulation of effector encoding genes by the UPR regulator Cib1 and identified the genes encoding the previously described Pit2 and Tin1-1 effectors as *bona fide* UPR targets. We observed that secretion of Pit2 is strongly increased under ER stress and a direct binding of Cib1 to the UPRE containing promoter fragments of both genes. Moreover, the targeted deletion of the UPRE abolished Cib1-dependent expression of *pit2* and resulted in significantly reduced virulence.

## Materials and Methods

### Strains and growth conditions

*Escherichia coli* strain TOP10 (Invitrogen) was used for cloning purposes and amplification of plasmid DNA. *U*. *maydis* cells were grown at 28°C in YEPSlight [37], complete medium (CM) [38] or yeast nitrogen base (YNB) medium [39,40] supplemented with 1% (w/v) glucose. UPR was induced by addition of 3 mM dithiothreitol (DTT) or 5 μg/ml tunicamycin (TM) (Sigma-Aldrich). ER stress assays were performed as described before [31]. All *U*. *maydis* strains used in this study are derived from the haploid pathogenic SG200 strain [9] and listed in S1 Table.

### *In silico* prediction of unfolded protein response elements

For motif search, UPRE1 and UPRE2 position weight matrices (PWM) were constructed according to the distribution matrix in [41]. Genes were considered as motif positive if they had at least one hit with more than 85% of the maximum PWM score in their promoter. Promoters were defined as 1 kb upstream regions of the predicted translation start site. Upstream sequences of 385 genes predicted to encode secreted proteins without enzymatic function [42] were extracted from Munich Information Center for Protein Sequences *Ustilago maydis* Data base (MUMDB) ftp://ftpmips.gsf.de/fungi/Ustilaginaceae/Ustilago_maydis_521/. Positive hits with a predicted function in the ER were excluded from downstream analysis.

### DNA and RNA procedures

Molecular methods followed the protocols of [43]. *U*. *maydis* DNA isolation and transformation procedures were performed according to [32]. For transformation, either linearized plasmid DNA or PCR generated linear DNA was used. All primers used in this study are listed in S2 Table. All plasmids were sequence verified prior transformation. Homologous integration of constructs was verified by PCR and Southern hybridization.

For gene deletions, a PCR-based approach was used [44]. Deletion strains of *cib1* were generated as described previously [34]. For the *cib1-3xHA* fusion, we used plasmid pCib3eGFP [34] and replaced the *Sfi*I flanked 3xeGFP-Hyg^R^ fragment with an *Sfi*I 3xHA-Hyg^R^ fragment of pUMa792 [45], generating plasmid pCib1-3xHA. For analysis of ΔUPRE functionality the *pit1/2* locus was first replaced by homologous recombination with the nourseothricin resistance cassette (Nat^R^) according to [46]. The resulting strain SG200Δ*pit1/2* was transformed with plasmid pPit1/2 or plasmid pPit1/2ΔUPRE containing 1 kb of the 3' region of the *pit1* ORF, the 3.8 kb *pit1/2* locus (consisting of *pit1*, *pit2* ORFs and promoter) (LB), a FRT-flanked Hyg^R^-cassette [47] and the 1 kb 3' region of *pit2* (RB), leading to replacement of the Nat^R^ cassette and reconstitution of the *pit1/2* locus with or without the predicted UPRE. Deletion of the predicted UPRE in the *pit1/2* promoter in plasmid pPit1/2ΔUPRE was generated by standard PCR techniques. The FRT-flanked Hyg^R^ cassette was excised from the genome using FLP recombinase as described in [47].

RNA extraction was performed as described before using Trizol reagent (Invitrogen) according to the manufacturer's instructions [35]. Integrity of isolated RNA was checked by ethidium bromide staining or by Bioanalyzer with an RNA 600 Nano LabChip kit (Agilent).

### Quantitative RT-PCR (qRT-PCR) analysis

qRT-PCR analysis was performed essentially as described before [31] with minor modifications. For screening of UPR-regulated effector gene expression, mRNA isolated from three biological replicates was pooled, subjected to cDNA synthesis and analyzed in two technical repeats. All other qRT-PCR experiments were conducted with three biological and two technical repeats thereof. qPCR was performed on a CFX-connect PCR cycler (BioRad) and statistical significance was calculated using Student's *t*-test.

### Quantitative chromatin immunoprecipitation (qChIP)

qChIP analysis was done essentially as described before [33]. Briefly, 50 ml cultures of *U*. *maydis* (SG200 *cib1*:*3xHA*) were grown in CM liquid medium to an OD_600_ = 0.6–0.8 and treated with 3 mM (f.c.) DTT to induce the UPR. After 3h cells were fixed with formaldehyde (f.c. 1%) for 15 min at room temperature (RT). The reaction was quenched by addition of 2.5 M Glycin (f.c. 125 mM). Cross-linked cells were harvested by centrifugation and washed three times with TBS (50 mM Tris, 150 mM NaCl) and resuspended in 1,5 ml FA-Lysis Buffer (50 mM HEPES-KOH pH 7.5, 150 mM NaCl, 1 mM EDTA, 1% Triton-X-100, 0.1% (w/v) sodium deoxycholate, 0.1% SDS) supplemented with 1x cOmplete EDTA-free (Roche) protease inhibitor cocktail. Cells were shock-frozen in liquid nitrogen and disrupted in a cell mill (Retsch MM200, 25Hz, 5min). Chromatin was sheared in a Covaris S200 set to yield a DNA average size of 300–500 bp. After centrifugation (17000g 15 min. 4°C) 400 μl chromatin solution and 30 μl monoclonal Anti-HA-Agarose (clone HA-7, Sigma-Aldrich) were incubated overnight on a rotating wheel at 4°C. 50μl of the chromatin was used as input control. The beads were washed twice with 500 μl FA lysis buffer, twice with 500 μl FA lysis high salt buffer (50 mM HEPES-KOH pH 7.5, 500 mM NaCl, 1 mM EDTA, 1% Triton-X-100, 0.1% (w/v) sodium deoxycholate, 0.10% SDS), twice with chromatin immunoprecipitation (ChIP) wash buffer (10 mM Tris-Cl pH 7.5, 250 mM LiCl, 1 mM EDTA, 0.5% Nonidet P40, 0.5% sodium deoxycholate), and once with 500μl TE (Tris-EDTA pH 7.5). The Protein-DNA complexes were eluted two times, once with 100μl ChIP elution buffer (50 mM Tris-Cl pH 7.5, 1 mM EDTA, 1% SDS) for 15 min at 65°C and once with 150 μl TE 0.67% SDS for 10 min at 65°C. The eluted samples were incubated over night at 65°C to reverse the crosslinks. After RNase A (0.8 mg/ml) incubation for 30 min at 37°C and Proteinase K (0.6 mg/ml) treatment for 2 h at 37°C DNA was recovered by column purification (PCR Purification Kit, Qiagen) and subjected to qPCR. Samples were analyzed on a BioRAD CFX connect PCR cycler using 1 μl of the precipitated or 1/100 diluted input DNA. Amplicons were normalized to the input control using the BioRad CFX manager software. Enrichment relative to input DNA was calculated according to https://www.thermofisher.com/de/de/home/life-science/epigenetics-noncoding-rna-research/chromatin-remodeling/chromatin-immunoprecipitation-chip/chip-analysis.html.

### Plant infection studies

The maize (*Zea mays*) cultivar VA35 was used for infection experiments. *U*. *maydis* strains were incubated at 28°C to an OD_600_ of 0.8–1.0, washed with H_2_O and concentrated to OD_600_ 1.0 in H_2_O. 0.5 ml of the cell suspension were injected into the basal stem of seven day-old VA35 maize seedlings. Two independent experiments were performed for each plant infection and the average scores for each symptom are shown in the respective diagrams. Symptoms were scored according to disease rating criteria reported by [9].

### Protein procedures

Secretion assays were performed essentially as described before [36]. Briefly, *U*. *maydis* cells were grown in CM liquid medium to an OD_600_ of 0.4. ER stress was induced by addition of 3 mM DTT (f.c) and cells were grown for additional 4 h. The cell pellet was isolated by centrifugation (3000g, 5 min, 4°C), resuspended in 1x SDS sample buffer and supplemented with glass beads, homogenized on a vibrax rotary shaker (IKA), boiled at 95°C for 10 min and subjected to SDS-PAGE analysis. Proteins in the culture supernatant were isolated by TCA precipitation. Briefly, TCA precipitated and acetone-washed protein pellets were resuspended in an appropriate volume of 1x SDS sample buffer (buffer volume was adjusted to cell numbers of individual cultures) and subjected to SDS-PAGE analysis. Immunoblot analysis was performed according to [48]. For detection of mCherry-tagged proteins commercially available monoclonal RFP antibody (6G6, Chromotek) was used at a 1:1000 dilution. HRP-conjugated anti-mouse immunoglobulin G (Promega) was used as secondary antibody and the Luminata Crescendo Western HRP Substrate (Merck Millipore) was employed for protein detection.

### Statistical analysis

Statistical significance was calculated using Student's *t*-test. In plant infection experiments statistical significance was calculated using the Wilcoxon-rank-sum test as described previously [39]. Results were considered significant if the *p*-value was < 0.05.

### Accession numbers

Sequence data from this article can be found at the Munich Information Center for Protein Sequences *Ustilago maydis* database (http://mips.helmholtz-muenchen.de/genre/proj/ustilago/) and the National Center for Biotechnology Information database under the following accession numbers: *actin* (*UMAG_11323*; XP_762364), *bip1* (*UMAG*_10534; XP_756724.1) *cib1* (*UMAG_11782*; XP_758585), *eIF2b* (XP_759656; *UMAG_04869*), *pit1* (XP_011387263.1; *UMAG_01374*), *pit2* (XP_011387264.1; *UMAG_01375*), *tin1-1* (XP_011392010.1; *UMAG_05294*).

## Results

### Identification of UPR-regulated effector genes

Previous studies revealed that in *U*. *maydis* the UPR regulates the pathogenic program to align the developmental progression and effector secretion during biotrophic growth *in planta*. Strains deleted for the Hac1-like UPR regulator Cib1 fail to proliferate after plant penetration and elicit plant defense responses that are not observed in wild-type infections, indicating that effector secretion might be compromised [31]. Since the UPR shows a multilayered crosstalk with pathways known to regulate expression of candidate effector genes, we asked whether the UPR not only affects the secretion of effector proteins, but might be as well involved in the transcriptional control of effector genes.

We have previously demonstrated that expression of *U*. *maydis* Cib1 suppressed ER stress sensitivity of *Saccharomyces cerevisiae HAC1* deletion strains [31], suggesting that Cib1 is able to bind to the same or similar potential unfolded protein response elements (UPREs) that are bound by Hac1. We used the distribution matrices that are based on UPRE1 or UPRE2 consensus sites (GGACAGCGTGTCG, CTACGTGTCT) generated by [41] to predict putative UPREs in the promoter region (1 kb upstream of the open reading frame (ORF)) of effector gene candidates in *U*. *maydis*. We analyzed the upstream regions of 385 genes predicted to encode secreted effector proteins without enzymatic functions [42]. Excluding candidates with a predicted UPR-related function and applying a minimum UPRE score of 0.85, we identified 74 genes harboring putative UPREs in their promoter regions (S3 Table). These candidates include the previously studied *pit2 (UMAG_01375)* and *pep1 (UMAG_01987)* genes [11–14] and several genes belonging to effector gene clusters 2B (*UMAG_01302*), 6A (*UMAG_02535*, *UMAG_02537*), 10A, (*UMAG_03748*, *UMAG_03750*), 19A (*UMAG_05294 UMAG_05299*, *UMAG_05309*, *UMAG_10555*) as well as the *mig2_1* (*UMAG_06178*), *mig2_2* (*UMAG_06179)* and *mig2_3* (*UMAG_1250*) genes [9,49–51].

We conducted a quantitative RT-PCR based screen to test for UPR-dependent expression of the candidate genes in the haploid pathogenic SG200 strain (WT). Albeit having a UPRE score below 0.85 we also included the previously characterized effector genes *tin2 (UMAG_05302*, UPRE score 0.708) and *cmu1 (UMAG_05731*, UPRE score 0.845) [16,52]. The conserved UPR marker gene *bip1* (*UMAG_15034*, UPRE score 0.859) served as positive control. Genes were considered to be UPR-regulated if gene expression was increased at least two-fold by treatment with the ER stress inducing agents dithiothreitol (DTT, 3 mM, 3 h) and tunicamycin (TM, 5 μg/ml, 4 h) in comparison to the untreated control and the Δ*cib1* control strain under the same conditions. While 66 of 76 genes tested did not show an ER stress dependent gene induction, significant *cib1*-dependent increase of *pep1*, *UMAG_03313*, *mig2_2*, *UMAG_06255*, *UMAG_10555* and *UMAG_11002* expression was found in response to DTT, but not TM. Expression of genes *UMAG_01750* and *UMAG_02535* was *cib1*-dependently increased only upon TM treatment. Moreover, *pit2* and *tin1-1* showed *cib1*-dependently induced expression under both, DTT- and TM-induced ER stress conditions (S3 and S4 Tables). For this reason, *pit2* and *tin1-1* were considered as the most promising candidates to further address a putative UPR-dependent effector gene expression.

### Cib1 regulates and directly binds to the promoter of *tin1-1*, *pit1* and *pit2*

**Fig 1:**
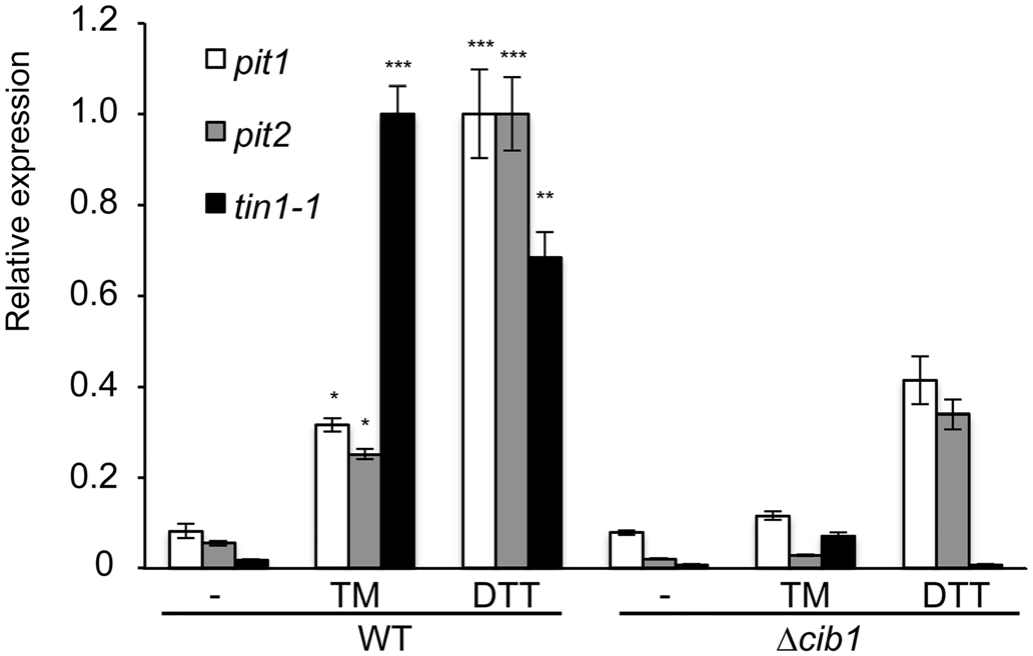
qRT-PCR analysis of UPR-dependent expression of *pit1*, *pit2* and *tin1-1*. RNA was prepared from exponentially growing *U*. *maydis* strains SG200 (WT) and *cib1* deletion (*Δcib1*) in YNB liquid medium supplemented with 5 μg/ml TM or 3 mM DTT. Expression of *pit1* and the effector genes *pit2* and *tin1-1* was measured in response to UPR induction. Expression values represent the mean of three biological replicates with two technical duplicates each. Error bars represent the standard error (SE). *indicates p-value < 0.05; **< 0.01 and ***< 0.001, respectively.

*tin1-1* is part of the effector gene cluster 19A, which contains 24 predicted effector genes. Deletion of the entire gene cluster almost completely abolished tumor formation [9]. However, deletion of a *tin1-1* containing sub-fragment of cluster 19A had only a minor effect on pathogenicity [49]. *pit2* encodes a secreted cysteine-protease inhibitor that is genetically linked to *pit1* (encoding a trans-membrane protein of unknown function). Both genes are highly upregulated during the infection process and crucial for pathogenic development [11]. Since expression of *pit1* and *pit2* is driven by a putatively bi-directional promoter, we speculated that *pit1* expression might be as well affected by ER stress. By qRT-PCR analysis, UPR-dependent gene expression of *tin1-1*, *pit1* and *pit2* was tested (Fig 1). We further included *bip1* and *actin* as positive and negative control to test assay conditions (S1 Fig). This showed significant upregulation of *tin1-1*, *pit1* and *pit2* in response to ER stress (Fig 1). Induced expression of all three genes was dependent on the presence of *cib1*. Interestingly, the predicted UPRE in the *pit1/2* promoter region is located in approximately the same distance between *pit1* and *pit2* ORFs, suggesting that a common regulatory element might mediate induction of both genes.

**Fig 2:**
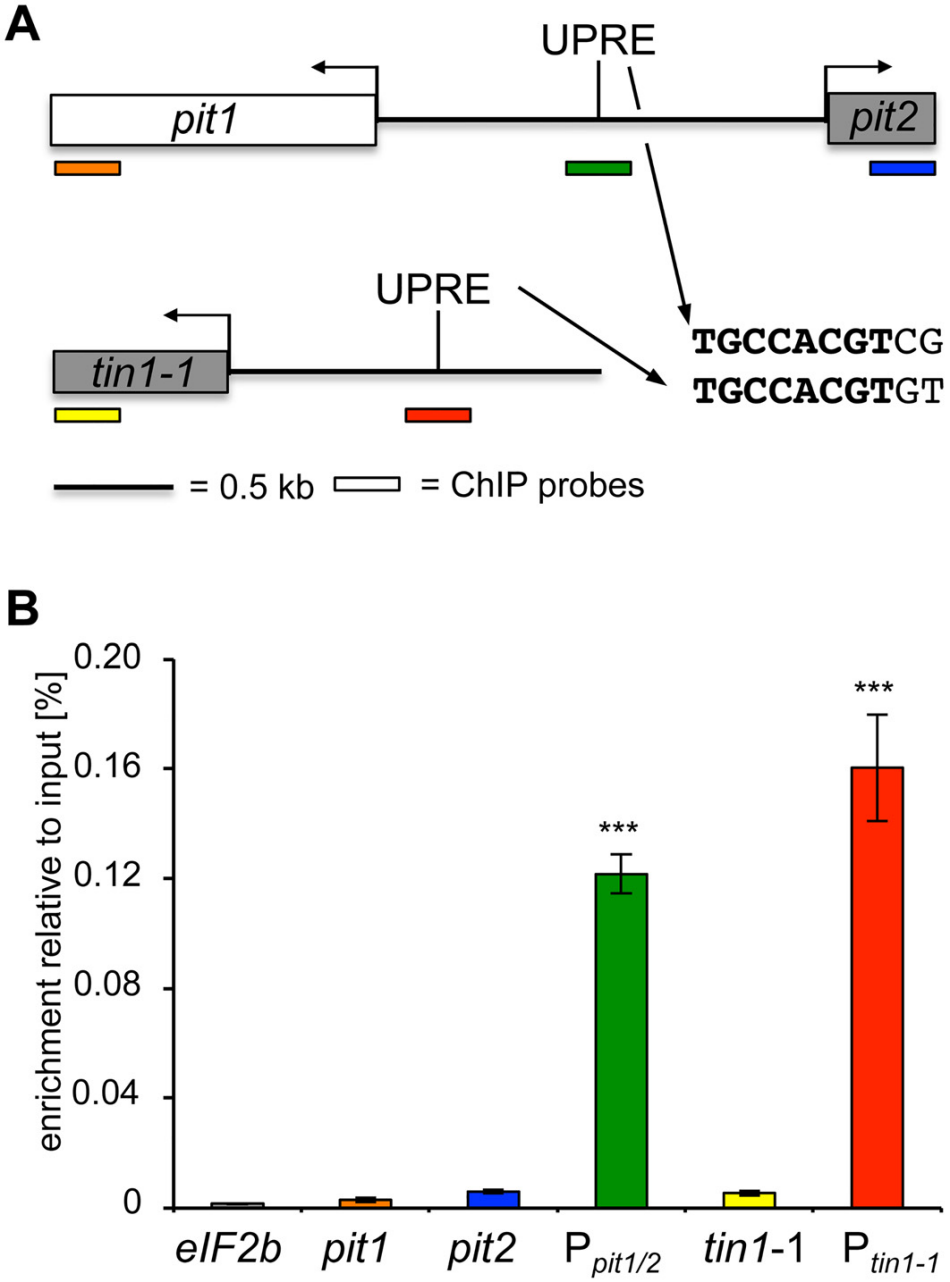
qChIP analysis of Cib1 binding to the *pit1/2*- and *tin1-1*-promoter. (A) Schematic overview of promoter organization and probe regions used for qChIP experiments. Sequence of the predicted Cib1 binding sites (UPRE) in the *pit1/2* and *tin1-1* promoter region is given in bold in case of identical nucleotides in *pit1/2* and *tin1-1* UPREs. (B) qChIP analysis of Cib1 binding to the *pit1/2* and *tin1-1* promoter in strain SG200*cib1-3xHA* 3 h after DTT (3 mM) treatment. The HA-tagged Cib1 protein was immunoprecipitated with anti HA-antibody coupled agarose beads (Sigma). Enrichment of immunoprecipitated DNA is shown relative to the input control. PCR-amplicons corresponding to the *pit1/2* and *tin1-1* promoter are significantly enriched compared to ORF controls. No significant enrichment was observed for PCR-amplicons corresponding to *pit1*, *pit2* and *tin1-1* ORFs in comparison to the *eIF2b* control. Given are the mean values of four independent experiments. Error bars represent the standard error (SE). Statistical significance was tested using Student's *t*-test. *indicates p-value < 0.05; **< 0.01 and ***< 0.001, respectively.

To address whether Cib1 directly regulates expression of *pit1*, *pit2* and *tin1-1*, we investigated binding of Cib1 to the predicted UPREs in respective promoter regions by quantitative chromatin immunoprecipitation (qChIP) analysis. To this end, strain SG200*cib1-3xHA* was generated for expression of a Cib1-3xHA fusion protein under the control of its endogenous promoter. To facilitate expression from the native genomic locus, the construct was integrated by homologous recombination, replacing the endogenous *cib1* gene. ER stress sensitivity of SG200*cib1-3xHA* was indistinguishable from the SG200 progenitor strain, demonstrating functionality of the fusion protein (S2 Fig). Enrichment was quantified relative to the input control for UPRE containing promoter regions, as well as for fragments corresponding to the ORF regions of *pit1*, *pit2*, *tin1-1*, and of *eIF2b* (Fig 2A) that is not regulated by the UPR. We observed a significant enrichment of fragments corresponding to UPRE containing promoter regions of *pit1/2* and *tin1-1*, whereas no enrichment was observed for regions corresponding to the respective ORFs (Fig 2B). In comparison to the *eIF2b* control, UPRE containing promoter fragments of *pit1/2* and *tin1-1* were 74.9 fold (+/- 7.8) and 95.1 fold (+/- 11.7) enriched, respectively. The predicted UPREs in the promoter of *pit1/2* and *tin1-1* share a consensus of TGCCACGT followed by CG or GT, respectively. This indicates that Cib1 regulates expression of *pit1*, *pit2* and *tin1-1* by direct binding to the promoter of these genes.

### The *pit1/2* UPRE is required for UPR-dependent gene expression and virulence of *U*. *maydis*

**Fig 3:**
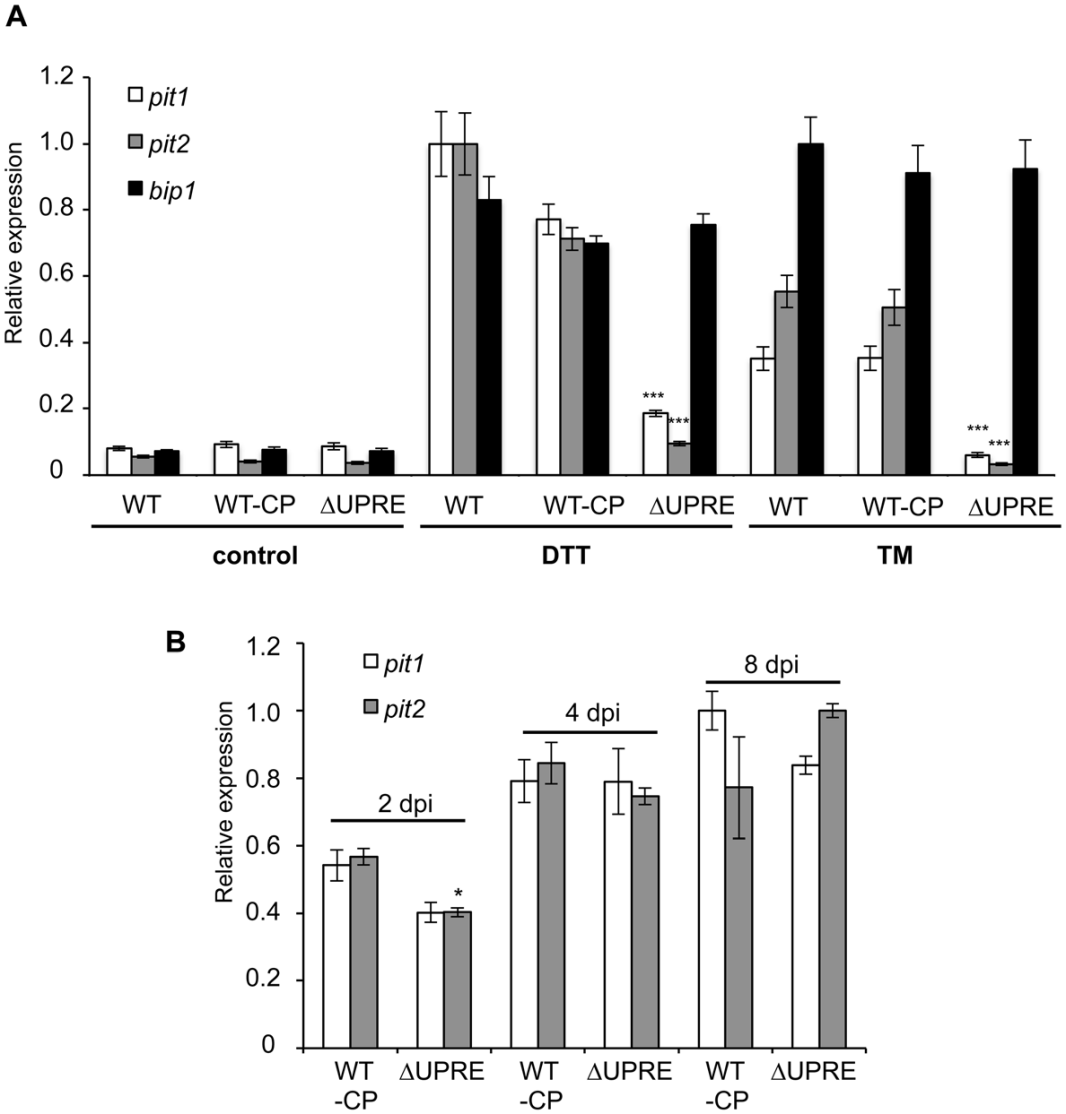
UPR-dependent *pit1* and *pit2* expression requires UPRE. (A) qRT-PCR analysis of UPR-dependent *pit1*, *pit2* and *bip1* expression. RNA was prepared from exponentially growing *U*. *maydis* strains SG200 (WT) and derivatives in YNB liquid medium supplemented with 5 μg/ml TM or 3 mM DTT. We tested two different complementation strains with (WT-CP) or without (ΔUPRE) the predicted UPR element in the *pit1/2*-promoter. Expression values represent the mean of three biological replicates with two technical duplicates each. Error bars represent the standard error (SE). Statistical significance was calculated using Students *t*-test. *indicates p-value < 0.05; **< 0.01 and ***< 0.001, respectively. (B) qRT-PCR analysis of infected maize leaves at 2, 4 and 8 days post inoculation (dpi). Maize seedlings were inoculated with equal cell numbers of indicated strains. RNA was prepared from three independent samples. Statistical significance was calculated using Students *t*-test. * indicates a p-value < 0.05.

We focused on the functional analysis of the *pit1/2* UPRE, since deletion of *pit1/2* leads to almost complete loss of *U*. *maydis* virulence, which allows functional readout in infection experiments. To test whether the predicted UPRE is required for UPR-dependent expression of *pit1/2* genes, the predicted UPRE motif (TGCCACGTCG) in the *pit1/2* promoter was deleted. To this end we first replaced the *pit1/2* locus with a nourseothricin resistance cassette, generating strain SG200Δ*pit1/2*. Deletion of the *pit1/2* locus had no effect on ER stress resistance (S2 Fig), indicating that *pit1/2* function is not related to the ER stress response in general. Subsequently the full *pit1/2* locus (including *pit1* and *pit2* ORFs and promoter), with or without the predicted UPRE, was re-integrated into the native locus (for details see material and methods). The resulting strains SG200-*pit1/2* (WT-CP) and SG200-*pit1/2ΔUPRE* (ΔUPRE) did not show alterations in basal expression levels of *pit1* and *pit2* in comparison to the SG200 (WT) progenitor strain. Moreover, when tested under DTT- or TM-induced ER stress conditions, we observed a robust induction of the conserved UPR target gene *bip1* in all three strains, indicating successful activation of the UPR. By contrast, ER stress induced *pit1* and *pit2* expression only in WT and WT-CP strains, whereas no induction was observed in the ΔUPRE strain (Fig 3A). This suggests that deletion of the UPRE in the *pit1/2* promoter abolished UPR-dependent induction of *pit1* and *pit2* expression.

**Fig 4:**
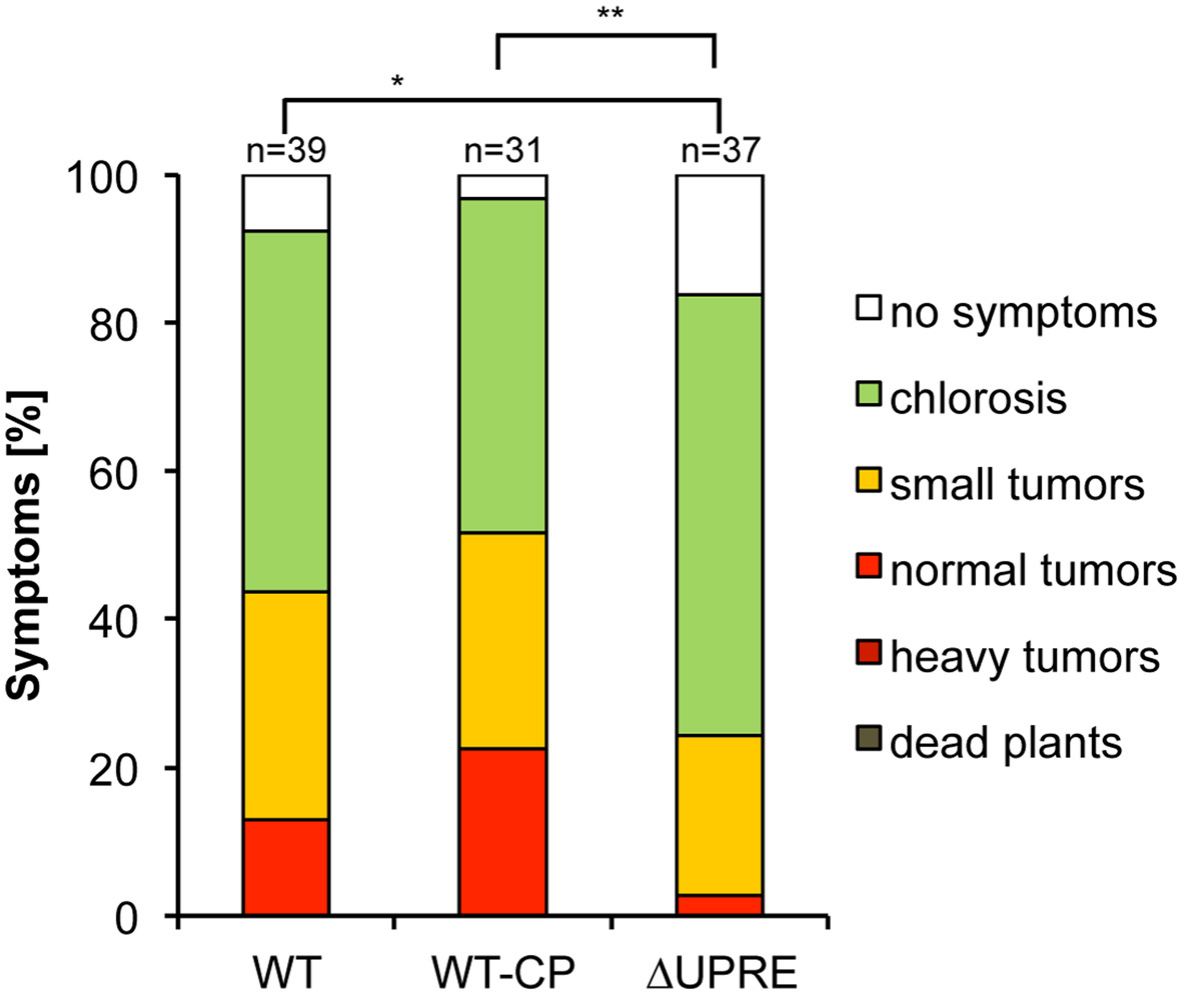
Loss of UPR-dependent *pit1/2* expression leads to significantly impaired virulence. The haploid pathogenic strain SG200 (WT) and derivatives were inoculated into seven-day-old VA35 maize seedlings. Comparison between WT and Δ*pit1/2* complemented strains harboring (WT-CP) or lacking the UPRE (ΔUPRE). Disease symptoms were rated eight days after inoculation and grouped into categories depicted on the right. n represents the number of inoculated plants. Statistical significance of alteration in disease rating was calculated using the Wilcoxon-rank-sum test. *P value < 0.05, **<0.01.

We next addressed expression levels of *pit1* and *pit2* in WT-CP and ΔUPRE strains during plant colonization. Quantification by qRT-PCR revealed reduced expression of *pit1/2* in ΔUPRE strains in comparison to the WT-CP control at 2 dpi, whereas at 4 and 8 dpi expression levels were almost identical to the control (Fig 3B). To test the impact of the *pit1/2* UPRE deletion on virulence plant infection experiments were performed. As expected deletion of *pit1/2* completely abolished virulence (S3 Fig). Importantly, virulence was fully restored in the SG200-*pit1/2* (WT-CP) complemented strain. By contrast, strain SG200-*pit1/2ΔUPRE* (ΔUPRE), lacking the UPRE motif showed significantly reduced virulence when compared to SG200 (WT) and SG200-*pit1/2* (WT-CP) (Fig 4A). This suggests, that UPR-dependent expression of *pit1/2* is required for full virulence of *U*. *maydis*.

### UPR activation results in increased secretion of Pit2

**Fig 5:**
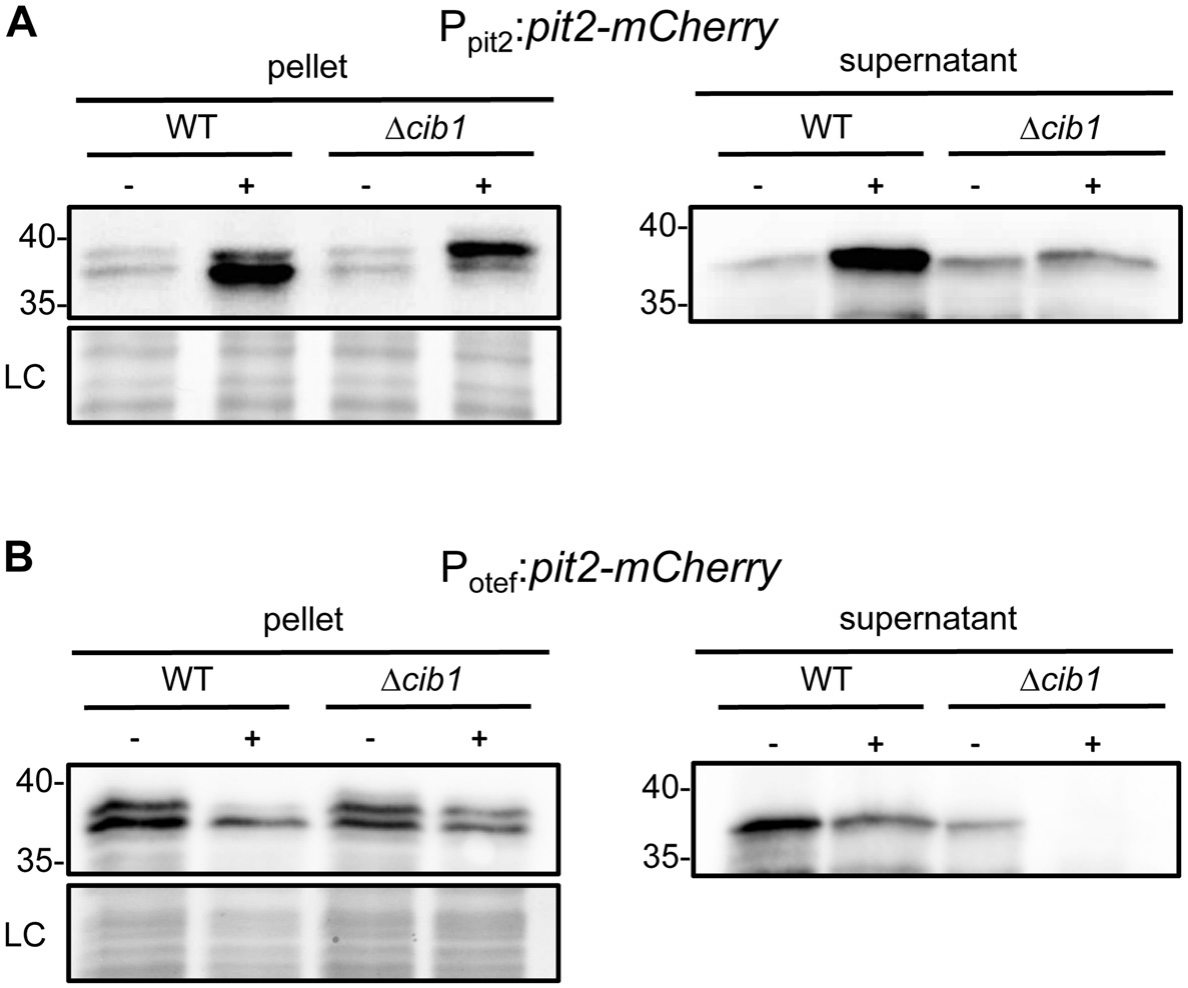
Secretion of Pit2-mCherry is strongly increased upon ER stress and dependent on Cib1. (A) The influence of DTT-mediated UPR activation on levels of Pit2-mCherry was investigated by immunoblot analysis using anti-mCherry antibodies in strains SG200Δ*pit2*-*pit2-mCherry* (WT) and the Δ*cib1* derivative were grown in CM liquid medium and treated with 3 mM DTT (+) to induce ER stress, or left untreated under otherwise identical conditions (-). After 4 h, cell pellets and supernatant were separated by centrifugation and proteins were subjected to SDS-PAGE analysis followed by immunodetection using anti-mCherry antibodies. Coomassie stained bands served as loading control (LC). (B) Posttranscriptional effects of an active UPR on Pit2-mCherry expression and secretion were monitored in strain SG200Δ*pit2*-*P*_*otef*_:*pit2-mCherry* (WT) and the Δ*cib1* derivative. Expression of *pit2-mcherry* is under control of the constitutively active *otef*-promoter. The experiment was performed as described for (A).

The unfolded protein response is supposed to be required for efficient secretion of effector proteins during biotrophic growth of *U*. *maydis* [31,36]. To determine the cumulative result of transcriptional and posttranscriptional effects of UPR activation on secretion of Pit2, we analyzed the amount of secreted Pit2-mCherry fusion protein in strain SG200Δ*pit2-pit2-mCherry* [11] and its Δ*cib1* derivative by immunoblot analysis. In these strains expression of Pit2-mCherry fusion protein is under control of its native promoter. Induction of DTT-mediated ER stress resulted in strongly increased levels of secreted Pit2-mCherry in the supernatant, compared to the untreated WT. In contrast, the level of secreted Pit2-mCherry was not affect by DTT treatment in the Δ*cib1* background (Fig 5A). Moreover, analysis of the pellet fraction revealed increased levels of Pit2-mCherry after DTT treatment in WT and to a lesser extent, also in Δ*cib1* strains (Fig 5A), which is in line with *pit2* expression levels under these conditions (Fig 1). Interestingly, in case of the Δ*cib1* strain, DTT-induced ER stress led to accumulation of a slightly higher migrating band that was not observed in the WT background or in the supernatant. Thus, our data indicates that UPR activation facilitates increased expression and secretion of Pit2-mCherry. However, assuming that the higher migrating band corresponds to the unprocessed Pit2-mCherry fusion protein including the 25 amino acid signal peptide (predicted molecular weight 39 kDa) and the lower migrating band to the processed form (predicted molecular weight 36 kDa), UPR might also be important for efficient processing and cleavage of the Pit2 signal peptide during ER stress.

To dissect transcriptional and posttranscriptional effects of UPR activation on Pit2 secretion, the Pit2-mCherry fusion was expressed under the control of the constitutive *otef* promoter (SG200Δ*pit2-P*_*otef*_:*pit2-mCherry*). Under these conditions, DTT-induced ER stress did not affect levels of Pit2-mCherry in the supernatant. By contrast, Pit2-mCherry was not detectable under these conditions in the Δ*cib1* background (Fig 5B). Also in the untreated control, Pit2-mCherry levels were lower in the supernatant of the Δ*cib1* strain in comparison to WT control. Analysis of the pellet fraction revealed no difference in intracellular levels of Pit2-mCherry independent of the genetic background (Fig 5B). However, similar to the experiments performed using the native *pit2* promoter, a higher migrating band was observed in untreated controls, which disappeared under ER stress in WT but not in the Δ*cib1* background. These results suggest an important role of the UPR for correct processing of Pit2-mCherry. However, high levels of secreted Pit2-mCherry require in addition UPR-dependent *pit2* gene expression.

## Discussion

UPR-dependent gene regulation is mediated by binding of the central UPR regulatory protein Hac1 to the UPREs in the promoter region of target genes. Recent studies in *S*. *cerevisiae* expanded the previously characterized UPRE sequence motif (GGACAGCGTG) [53] and identified a second motif, termed UPRE2 (CTACGTGTCT), that is bound by Hac1 *in vitro* and sufficient to mediate reporter gene expression *in vivo* [41]. In this study we performed an *in silico* prediction of UPREs in the promoter regions of *U*. *maydis* genes encoding potentially secreted effector proteins. Although promoters of 74 candidate effector genes carry predicted UPREs, only two genes showed UPR-dependent gene expression. When tested against previously published UPR target genes this approach identified putative UPREs in the promoter region of *bip1* (UPRE score 0.859), *lhs1* (UPRE score 0.961), *spp1* (UPRE score 0.911) and *dnj1* (UPRE score 0.866) [31,36]. This indicates that bioinformatic prediction of UPREs alone is not sufficient for the identification of UPR target genes and requires further experiments *in vivo* for verification.

Interestingly, the UPRE motif identified in the *pit1/2* promoter is also found in the promoter of *cib1* and *UMAG_01018* (encoding the *U*. *maydis* cyclophilin Cpr5 orthologue) genes, both of which are known UPR targets [31, 54]. This finding is consistent with a previously postulated positive autoregulation of Cib1 [31], and with studies in *S*. *cerevisiae* and *Aspergillus niger* showing that expression of *HAC1* and *hacA* is under positive autoregulation, respectively [55,56]. By contrast, the *tin1-1* UPRE was absent in all other promoters of the *U*. *maydis* genome. qChIP-analysis showed direct binding of Cib1 to the *pit1/2* promoter regions, and targeted deletion of the UPRE in the *pit1/2* promoter provided further support for a direct regulation of *pit1* and *pit2* by Cib1. To the best of our knowledge, neither a direct nor an indirect regulation of effector gene expression by Hac1-like UPR regulatory proteins has been described, yet. However, it remains to be determined, whether the UPRE is not only required but also sufficient for UPR-dependent gene regulation.

We did not observe significantly induced expression during ER stress of *pit1* and *pit2* orthologous genes in the related smut fungi *Sporisorium reilianum* and *Ustilago hordei* (not shown), indicating that regulation of *pit1/2* by the UPR might be an adaptation specific for *U*. *maydis*. The lifestyle of *S*. *reilianum* and *U*. *hordei* is considerably different compared to *U*. *maydis*. While *U*. *maydis*-induced symptoms like tumor development can occur on all aerial parts of the plant, spore formation of *S*. *reilianum* and *U*. *hordei* is restricted to the reproductive tissue of their host plants [57]. We thus cannot exclude the possibility that UPR-dependent regulation of effector genes also exists in *S*. *reilianium* or *U*. *hordei* but involves other target genes.

In *U*. *maydis*, expression of several effector genes is induced already prior to plant penetration [10,33], most of which are regulated by the C2H2 zinc finger and homeodomain transcription factors Biz1 and Hdp2, respectively [10,58]. Expression of both transcription factors is regulated by Rbf1, the master regulator of the *b*-dependent regulatory network, which in turn regulates the morphogenetic transition from yeast-like sporidial growth to the filamentous form that is infectious to the plant [33,34]. As our previous work demonstrated a suppressive effect of an active UPR on *rbf1* expression [31], effector gene regulation by Biz1 and Hdp2 might be as well negatively affected.

Deletion of the *pit1/2* UPRE fully abolished ER stress dependent gene induction and infection experiments revealed significantly reduced virulence of strains lacking the UPRE. Expression of *pit1* and *pit2* is also regulated by the homeodomain transcription factor Hdp2, that functions within the *b*-regulatory cascade. Thus, the redundant or compensatory gene regulation of *pit1/2* during pathogenic growth *in planta* might explain why expression levels of *pit1/2* were only reduced at 2 dpi.

Pit2 functions as a cysteine protease inhibitor and prevents salicylic acid induced cell death. It is tempting to speculate that UPR activity during biotrophic growth is not static but also influenced by plant-derived factors. In this way, UPR-dependent regulation of *pit2* expression might serve as a fine-tuning mechanism to quickly adapt *pit2* levels to the plant environment. By contrast, the function of Tin1-1 is not essential for virulence of *U*. *maydis* and *tin1-1* levels are highest at later stages of plant colonization [49]. A potential function in virulence might not be visible due to redundant protein functions. However, deletion of the whole *tin1* gene family (*UMAG_05294*, *UMAG_10554*, *UMAG_05295*, *UMAG_12302* and *UMAG_10553*) only slightly affected virulence under the conditions tested and the physiological role of UPR-dependent *tin1-1* regulation remains to be identified. Currently, we cannot rule out that other effector genes are also subject to UPR-regulation. Thus, it is possible that UPR-dependent effector gene expression has a quantitative effect on pathogenic development. Importantly, abolishment of a single regulatory connection between the UPR and *pit2* already affected virulence of *U*. *maydis*, indicating that a complete loss of UPR-dependent effector gene regulation might have even more dramatic effects.

Previous studies revealed that the ER co-chaperone Dnj1 is required for secretion of the chorismate mutase Cmu1 under ER stress conditions [36]. Consistently, ER stress strongly increased the levels of secreted Pit2 protein. However, not only secretion but also processing of Pit2 requires a functional UPR. Coupling of both pathways would reduce the risk of producing increased amounts of unprocessed or misfolded proteins in the ER, and thereby reduce the burden on cellular energy levels. Thus, it is likely that both, transcriptional and posttranscriptional effects of UPR activation facilitate the efficient secretion of Pit2 and eventually, also other effector proteins during biotrophic growth of *U*. *maydis*.

## Supporting Information

S1 Fig Positive and negative controls for qRT-PCR analysis of UPR-dependent expression. RNA was prepared from exponentially growing *U*. *maydis* strains SG200 (WT) and *cib1* deletion (*Δcib1*) in YNB liquid medium supplemented with 5 μ/ml TM or 3 mM DTT. Expression of *bip1* (positive control) and *actin* (negative control) was measured in response to UPR induction. Expression values represent the mean of three biological replicates with two technical duplicates each.
(TIF)

S2 Fig Deletion of *pit1* and *pit2* and expression of the Cib1-3xHA fusion protein does not affect ER stress resistance. ER stress assay of *U*. *maydis* strain SG200 and derivatives. Serial ten-fold dilutions of indicated strains were spotted on YNB solid medium supplemented with glucose. TM or DTT was used to induce ER stress. Plates were incubated for 48 hours at 28°C.
(TIF)

S3 Fig Deletion of *pit1/2* leads to complete loss of pathogenicity. The haploid pathogenic strain SG200 (WT) and the Δ*pit1/2* derivative were inoculated into seven-day-old VA35 maize seedlings. Disease symptoms were rated eight days after inoculation and grouped into categories depicted on the right. n represents the number of inoculated plants.
(TIF)

S1 Table Strains used in this study. (DOCX)

S2 Table Primers used in this study. (XLSX)

S3 Table Overview of candidate genes with predicted UPRE >0.85. (XLSX)

S4 Table Expression of candidate genes during ER stress screening by qRT-PCR. (XLSX)
