# Supplementary figures and images for: Unfolded Protein Response (UPR) Regulator Cib1 Controls Expression of Genes Encoding Secreted Virulence Factors in Ustilago maydis
Source: PLoS One. 2016 Apr 19;11(4):e0153861. doi: 10.1371/journal.pone.0153861 (PMC4836707; doi:10.1371/journal.pone.0153861)

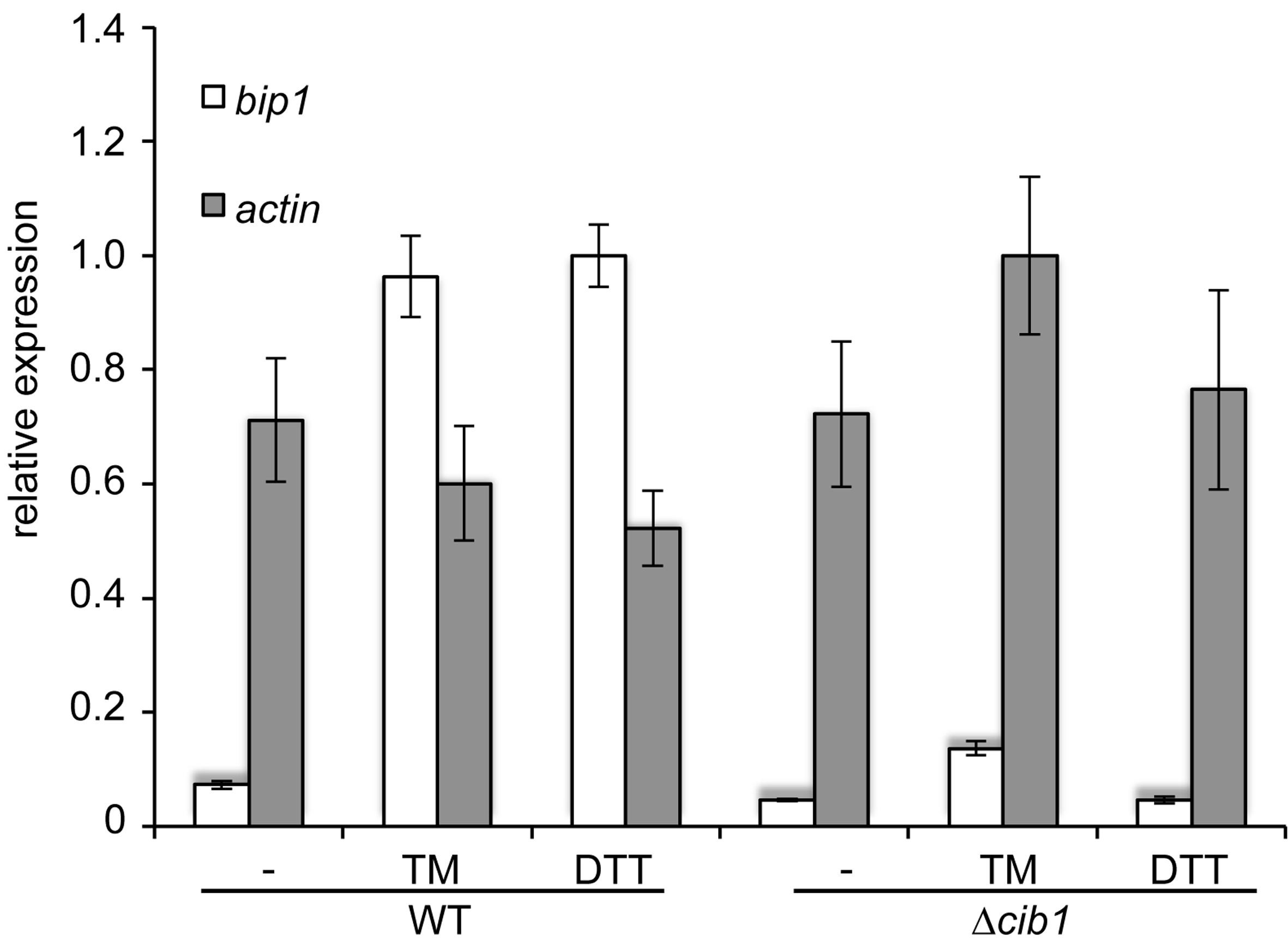

Supplement: S1 Fig — RNA was prepared from exponentially growing U. maydis strains SG200 (WT) and cib1 deletion (Δcib1) in YNB liquid medium supplemented with 5 μ/ml TM or 3 mM DTT. Expression of bip1 (positive control) and actin (negative control) was measured in response to UPR induction. Expression values represent the mean of three biological replicates with two technical duplicates each. (TIF) [file pone.0153861.s001.tif]

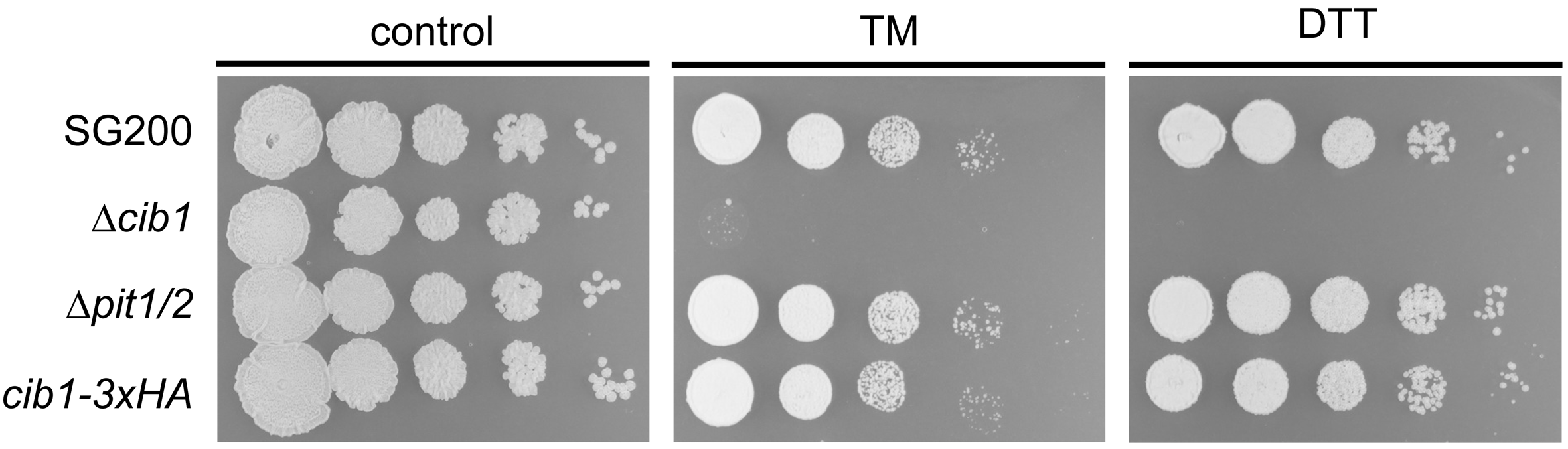

Supplement: S2 Fig — ER stress assay of U. maydis strain SG200 and derivatives. Serial ten-fold dilutions of indicated strains were spotted on YNB solid medium supplemented with glucose. TM or DTT was used to induce ER stress. Plates were incubated for 48 hours at 28°C. (TIF) [file pone.0153861.s002.tif]

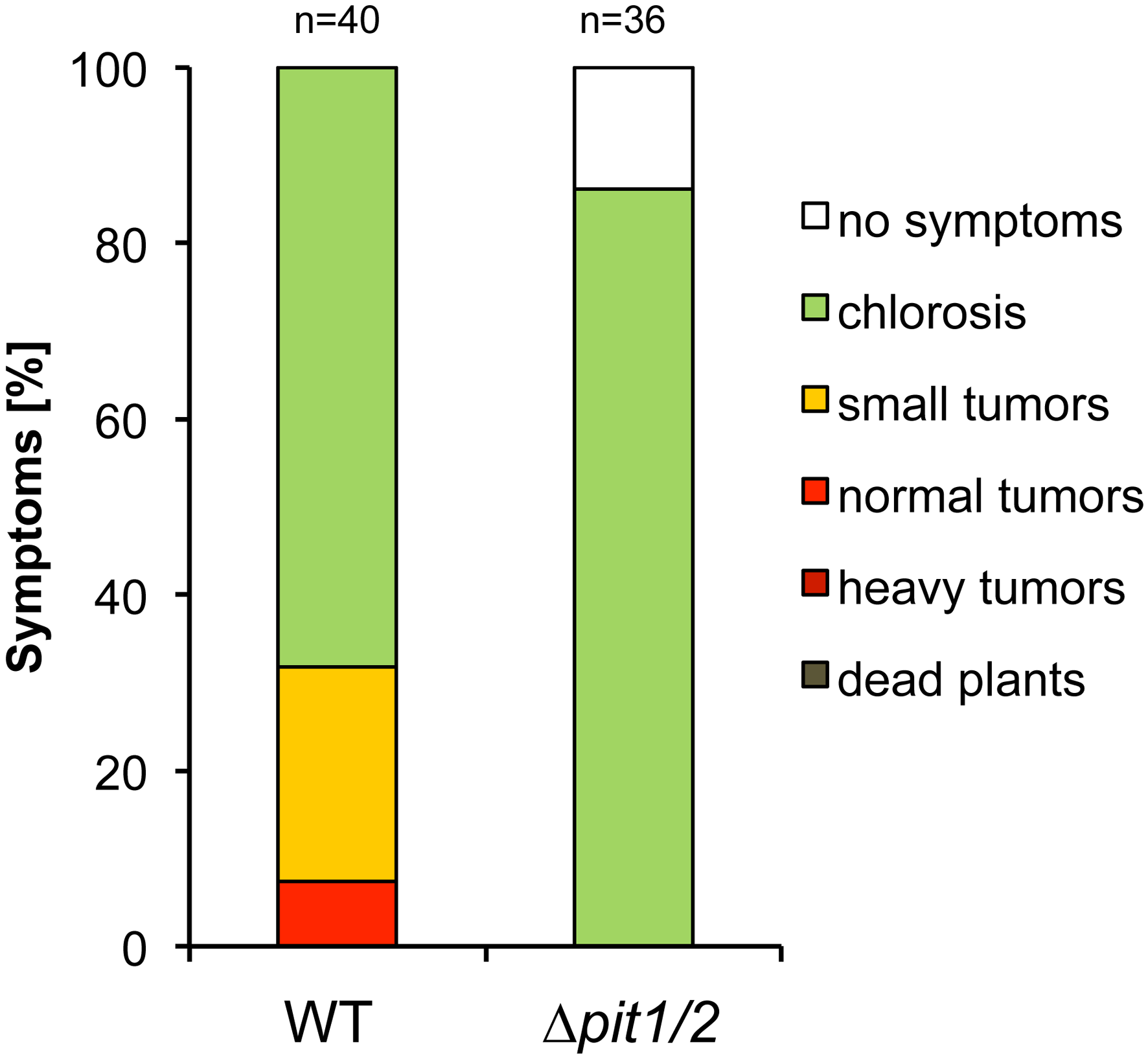

Supplement: S3 Fig — The haploid pathogenic strain SG200 (WT) and the Δpit1/2 derivative were inoculated into seven-day-old VA35 maize seedlings. Disease symptoms were rated eight days after inoculation and grouped into categories depicted on the right. n represents the number of inoculated plants. (TIF) [file pone.0153861.s003.tif]
