# Supplementary material for: Unfolded Protein Response (UPR) Regulator Cib1 Controls Expression of Genes Encoding Secreted Virulence Factors in Ustilago maydis
Source: PLoS One. 2016 Apr 19;11(4):e0153861. doi: 10.1371/journal.pone.0153861 (PMC4836707; doi:10.1371/journal.pone.0153861)
Supplement: S1 Table — (DOCX) [file pone.0153861.s004.docx]

**S1 Table: Strains used in this study**

| **Strain** | **Genotype** | **Reference** |
| --- | --- | --- |
| SG200 | *a1 mfa2 bE1bW2* | Kämper et al., 2006^(1)^ |
| SG200 ∆*cib1* | *a1 mfa2 bE1bW2* ∆*cib1* | Heimel et al., 2010^(2)^ |
| SG200 *cib1:3xHA Δcib1* | *a1 mfa2 bE1bW2* ∆*cib1 cib1:3xHA* | this study |
| SG200 ∆*pit1/2* | *a1 mfa2 bE1bW2* ∆*pit1/2* | this study |
| SG200 ∆*pit1/2 P_wt_:pit1/2* | *a1 mfa2 bE1bW2* ∆*pit1/2 P_wt_:pit1/2* | this study |
| SG200 ∆*pit1/2 P_∆UPRE_:pit1/2* | *a1 mfa2 bE1bW2* ∆*pit1/2 P_∆UPRE_:pit1/2* | this study |
| SG200 ∆*pit2 P_pit2_-pit2-mCherry* | *a1mfa2 bW2bE1* ∆*pit2 ip^r^[P_pit2_:pit2:mcherry]ip^s^* | Doehlemann et al., 2011^(3)^ |
| SG200 ∆*pit2 ∆cib1 P_pit2_-pit2-mCherry* | *a1mfa2 bW2bE1* ∆*pit2* ∆*cib1 ip^r^[P_pit2_:pit2:mcherry]ip^s^* | this study |
| SG200 ∆*pit2* *P_otef_-pit2-mCherry* | *a1mfa2 bW2bE1* ∆*pit2 ip^r^[P_otef_:pit2:mcherry]ip^s^* | this study |
| SG200 ∆*pit2* ∆*cib1* *P_otef_-pit2-mCherry* | *a1mfa2 bW2bE1* ∆*pit2* ∆*cib1 ip^r^[P_otef_:pit2:mcherry]ip^s^* | this study |

**(1) Kämper, J., Kahmann, R., Bölker, M., Ma, L.J., Brefort, T., Saville, B.J., Banuett, F., Kronstad, J.W., Gold, S.E., et al.** (2006). Insights from the genome of the biotrophic fungal plant pathogen *Ustilago maydis.* Nature **444,** 97-101.  **(2) Heimel, K., Scherer, M., Schuler, D., and Kämper, J.** (2010a). The *Ustilago maydis* Clp1 protein orchestrates pheromone and b-dependent signaling pathways to coordinate the cell cycle and pathogenic development. The Plant cell **22,** 2908-2922. **(3)** **Doehlemann, G., Reissmann, S., Assmann, D., Fleckenstein, M., Kahmann, R.** (2011). Two linked genes encoding a secreted effector and a membrane protein are essential for *Ustilago maydis*-induced tumour formation. Mol Microbiol. **81**, 751-66.
